# High-Quality Draft Genome Sequence of Fusarium oxysporum f. sp. *cubense* Strain 160527, a Causal Agent of Panama Disease

**DOI:** 10.1128/MRA.00654-19

**Published:** 2019-07-18

**Authors:** Shuta Asai, Yu Ayukawa, Pamela Gan, Sachiko Masuda, Ken Komatsu, Ken Shirasu, Tsutomu Arie

**Affiliations:** a Center for Sustainable Resource Science, RIKEN, Yokohama, Kanagawa, Japan; b PRESTO, Japan Science and Technology Agency, Kawaguchi, Japan; c United Graduate School of Agricultural Science, Tokyo University of Agriculture and Technology (TUAT), Fuchu, Tokyo, Japan; Broad Institute

## Abstract

Fusarium oxysporum f. sp. *cubense* is the causal agent of banana Fusarium wilt, also known as Panama disease. Here, we present a high-quality genome sequence of F. oxysporum f. sp. *cubense* strain 160527. The genome assembly is composed of 12 contigs with a total assembly length of 51,139,495 bp (*N*_50_ contig length, 4,884,632 bp).

## ANNOUNCEMENT

Fusarium oxysporum f. sp. *cubense* is the causal agent of banana Fusarium wilt, also known as Panama disease. Based on the pathogenicity to specific banana cultivars, F. oxysporum f. sp. *cubense* is classified into three races (race 1, race 2, and race 4) (1). The current epidemic in ‘Cavendish’ bananas is caused by F. oxysporum f. sp. *cubense* tropical race 4 (TR4). The threat of F. oxysporum f. sp. *cubense* TR4 to banana crops has gained global and scientific attention in recent years (1–3). In 2016, Panama disease was observed on ‘Shima-banana’ plants in Okinawa, Japan (4). This paper reports the genome sequence of F. oxysporum f. sp. *cubense* strain 160527, isolated from the infected ‘Shima-banana’ plants in Okinawa.

Genomic DNA of F. oxysporum f. sp. *cubense* strain 160527 was extracted from hyphae grown in NO_3_ medium (0.17% yeast nitrogen base, 3% sucrose, and 100 mM KNO_3_) using cetyltrimethylammonium bromide (CTAB) and the Genomic-tip 100/G kit (Qiagen) as described for the 1,000 Fungal Genomes Project (5). The genome was sequenced using the PacBio Sequel system. PacBio libraries were prepared and size selected with a 30-kb cutoff using BluePippin (Sage Science, MA, USA). Sequencing of one single-molecule real-time (SMRT) cell was performed using Sequel sequencing chemistry v3.0. A total of 677,986 filtered subreads (*N*_50_ length of filtered subreads, 31,070 bp) were assembled using the Hierarchical Genome Assembly Process (HGAP) v4 within SMRT Link (v6.0.0). Default values were kept, with the expected genome size set to 60 Mb. The assembly yielded 12 contigs with a total assembly size of 51,139,495 bp (47.48% GC content, an *N*_50_ value of 4,884,632 bp, and a maximum contig size of 6,605,270 bp; Table 1). Completeness of gene space within the assembly was assessed through the presence of conserved single-copy genes using Benchmarking Universal Single-Copy Orthologs (BUSCO) v3.0.2 (6, 7). The analysis with the Sordariomycetes data set (3,725 genes) showed the presence of 3,691 genes (99.1%) in the assembly (Table 1). Out of 12 contigs, 8 contigs had ≥25 copies of the telomeric repeat unit CCCTAA within 50 kb of the end on both ends, whereas the other 4 contigs contained those copies on one end (Table 1), indicating that the assembly is of a very high quality at almost the chromosomal level.

**TABLE 1:** F. oxysporum f. sp. *cubense* strain 160527 genome statistics

Statistic	Value
Assembly	
Total coverage (fold)	246
Assembly size (bp)	51,139,495
No. of contigs	12
Maximum contig length (bp)	6,605,270
*N*_50_ contig length (bp)	4,884,632
GC content (%)	47.48
BUSCO coverage (%)	99.1
Presence of telomeric repeats	
On both ends	8
On one end	4
None	0
Gene model	
Total no. of genes	16,536
Total no. of proteins	16,784

For gene prediction, published RNA sequencing data (8) were aligned to the genome using HISAT2 v2.1.0 (9) and used to guide gene model prediction using the BRAKER1 v1.9 pipeline (10). BRAKER1 was run with the repeat-soft-masked genome created by RepeatMasker v4.0.7 (with the settings -engine ncbi -species “ascomycota” –xsmall; http://www.repeatmasker.org/), using the fungal and soft-masking options. A total of 16,536 genes encoding 16,784 proteins were predicted (Table 1). The larger number of proteins than genes is the result of multiple transcripts predicted for some genes. Gene-coding sequences were annotated using InterProScan v5.35-74.0 (11) and through BLASTp (E value, <1 × 10^−6^) searches against the July 2018 release of the SWISS-PROT database (12). BLASTx analyses (E value, <1 × 10^−6^) for the 14 known secreted-in-xylem genes (SIX1 to SIX14) (13, 14) identified homologs of SIX1 (two homologs), SIX6 (two homologs), SIX9 (one homolog), and SIX13 (one homolog) on contig 2. Based on variation of the SIX gene profile in F. oxysporum f. sp. *cubense* races previously reported (15), F. oxysporum f. sp. *cubense* strain 160527 seems to belong to race 1.

### Data availability.

This whole-genome shotgun project has been deposited in DDBJ/ENA/GenBank under the accession number SRMI00000000 (BioProject number PRJNA529756, BioSample number SAMN11282975, and SRA number SRR9090003). The version described in this paper is the first version, SRMI01000000.
